# Oropouche virus NSs protein suppresses host transcription by targeting the RNA polymerase II RPB1 protein

**DOI:** 10.1128/jvi.01176-25

**Published:** 2025-09-10

**Authors:** Eduardo Jurado-Cobena, Cigdem Alkan, Tetsuro Ikegami

**Affiliations:** 1 Department of Microbiology and Immunology, The University of Texas Medical Branch at Galveston 12338, Galveston, Texas, USA; 2 Department of Pathology, The University of Texas Medical Branch at Galveston 12338, Galveston, Texas, USA; 3 Sealy Institute for Vaccine Sciences, The University of Texas Medical Branch at Galveston 12338, Galveston, Texas, USA; 4 Center for Biodefense and Emerging Infectious Diseases, The University of Texas Medical Branch at Galveston 12338, Galveston, Texas, USA; University Medical Center Freiburg, Freiburg, Germany

**Keywords:** Oropouche virus, NSs protein, RNA polymerase II, Rift Valley fever virus MP-12 strain, nucleolus, NPM1, proteasomal degradation

## Abstract

Oropouche fever is a debilitating disease caused by Oropouche virus (OROV), an arthropod-borne member of the Peribunyaviridae family. Despite its public health significance, the molecular mechanisms driving OROV pathogenesis remain poorly understood. In other bunyaviruses, the nonstructural NSs protein encoded by the small (S) genome segment acts as a major virulence factor. In this study, infection with the OROV MD023 strain led to nuclear accumulation of NSs and redistribution of nucleophosmin 1 (NPM1) from the nucleolus. OROV infection suppressed nascent RNA synthesis and resulted in decreased levels of the RNA polymerase II (RNAP II) subunit RPB1, along with reduced phosphorylation of its C-terminal domain (CTD) at serine 2 and serine 5 residues. When expressed from a recombinant Rift Valley fever virus MP-12 strain, OROV NSs colocalized with NPM1 and contributed to its nucleolar redistribution. Furthermore, expression of OROV NSs induced a marked reduction in the hyperphosphorylated RNAP IIo form, which was largely restored upon treatment with the proteasome inhibitor MG132. These findings suggest that OROV NSs promote RNAP II degradation and suppress host transcription, underscoring its potential role in modulating host responses during infection.

**IMPORTANCE:**

Oropouche fever is a viral disease characterized by fever, headaches, and body aches, affecting thousands of people in tropical regions. The Oropouche virus (OROV) has caused and continues to cause medium to large-scale outbreaks, highlighting the urgent need to better understand its basic biology. This study focused on the viral NSs protein, which modulates host antiviral responses. Our findings demonstrate that NSs disrupt RNA polymerase II, a key enzyme in host gene expression, by reducing its activity and stability. Additionally, OROV infection alters the nucleolus, a critical center for cellular stress responses and ribosome biogenesis. These disruptions suggest that OROV suppresses host transcription and nucleolar function, thereby impairing the cellular antiviral response. Understanding these mechanisms provides new insights into host-virus interactions and viral strategies for modulating host cell responses.

## INTRODUCTION

Oropouche fever is a dengue-like acute febrile illness with high morbidity, characterized by a biphasic fever, severe headache, and, in some cases, meningitis or meningoencephalitis (1, 2). The causative agent of Oropouche fever is *Orthobunyavirus oropoucheense* (Oropouche virus, OROV), a member of the genus *Orthobunyavirus* within the family Peribunyaviridae (3). Although OROV has been isolated from sylvatic mosquitoes such as *Aedes serratus* and *Coquillettidia venezuelensis*, as well as from urban mosquitoes such as *Culex quinquefasciatus*, their roles as maintenance vectors remain inconclusive (4–6). Midges (*Culicoides paraensis*) are considered the principal vector of the epidemic urban cycle of OROV transmission (6). The first isolation of OROV was reported in 1955 from a forest worker in Vega de Oropouche, Trinidad (4). OROV caused significant outbreaks in the Amazon River Basin, particularly in Pará state, Brazil, in 1961. By 2000, more than 30 outbreaks had been documented across Brazil. Additional outbreaks were reported in northern and southern Peru starting in the 1990s and in Panama in 1989. It is estimated that over 500,000 individuals have been infected with OROV over the past 60 years. The most recent outbreak, ongoing since 2023, has spread to Brazil, Peru, Colombia, Bolivia, the Dominican Republic, and Cuba, with over 11,000 reported cases, including two fatalities and suspected vertical transmission in pregnant women (7, 8). While OROV has historically been considered a neglected tropical disease, the recent outbreak has highlighted its public health significance and the urgent need for effective countermeasures to mitigate the impact of future outbreaks.

OROV has a tripartite, single-stranded, negative-sense RNA genome consisting of small (S), medium (M), and large (L) segments. The L segment encodes the RNA-dependent RNA polymerase (L protein), the M segment encodes the two envelope glycoproteins (Gn and Gc) and a nonstructural NSm protein, and the S segment encodes genes for the nucleocapsid (N) protein and a small nonstructural NSs protein. The *N* and *NSs* genes of the S segment are encoded in overlapping reading frames. The NSs protein is a major virulence factor in many pathogenic bunyaviruses and plays a crucial role in suppressing host antiviral responses, including innate immunity (9–11).

As of 2024, the International Committee on Taxonomy of Viruses recognizes at least 134 species within the genus *Orthobunyavirus*, organized into at least 18 serogroups. These serogroups include Bunyamwera, which contains species such as *Orthobunyavirus bunyamweraense* (Bunyamwera virus, BUNV); California, which includes species like *Orthobunyavirus lacrosseense* (La Crosse virus, LACV); and Simbu, comprising species, including *Orthobunyavirus schmallenbergense* (Schmallenberg virus, SBV), *Orthobunyavirus akabaneense* (Akabane virus, AKAV), and OROV. Previous studies have revealed a similar function of bunyavirus NSs proteins in the nucleus. The BUNV NSs protein localizes to both the cytoplasm and nucleus (12) and inhibits the phosphorylation of the C-terminal domain (CTD) of RNA polymerase II (RNAP II) RPB1 subunit (13). The CTD comprises 52 repeats of the YSPTSPS sequence, with phosphorylation at serine 2 and serine 5 regulating mRNA elongation and 3′-end processing, respectively (14). Expression of BUNV NSs specifically inhibits serine 2 phosphorylation, and its interaction with the Mediator protein MED8 is likely involved in this regulatory process (15). Meanwhile, expression of LACV NSs proteins affects the phosphorylation of the RNAP II RPB1 CTD at both serine 2 and serine 5, leading to the degradation of the hyperphosphorylated form (IIo) (16). The SBV NSs proteins primarily localize to the nucleoli, and the nuclear stress triggered by NSs likely promotes the post-translational degradation of RNAP II RPB1 (17, 18).

It has been shown that OROV, but not a recombinant OROV lacking the *NSs* gene, can inhibit the induction of type I interferons in A549 cells (19). However, our understanding of OROV NSs proteins remains limited. Since OROV is closely related to SBV within the Simbu serogroup, we hypothesize that its NSs proteins share a similar biological function in modulating host transcription. In this study, we characterized the nuclear localization of OROV NSs proteins and their role in transcriptional suppression in infected cells.

## MATERIALS AND METHODS

### Media, cells, and viruses

Vero cells (ATCC CCL-81) were cultured in Dulbecco’s modified Eagle medium supplemented with 10% fetal bovine serum (FBS; Gibco), 100 U/mL penicillin, and 100 µg/mL streptomycin (Invitrogen) in a humidified cell culture incubator (5% CO_2_, 37°C). The OROV MD023 strain was kindly provided by the World Reference Center for Emerging Viruses and Arboviruses (WRCEVA) at the University of Texas Medical Branch (UTMB). The viral stock was generated from a plaque isolate obtained from Vero cells infected with the original stock virus. We generated recombinant Rift Valley fever virus (RVFV) MP-12 strains in which the RVFV NSs gene was replaced with Renilla luciferase (rLuc), OROV NSs, or OROV NSs fused to an epitope tag, either a Flag-tag (N-DYKDDDDK-C) at the N- or the C-terminus or a tandem Strep-tag II and Flag tag (SF-tag: N-LEWSHPQFEKGEDYKDDDDK-C, with the Strep-tag sequence underlined) at the C-terminus. To minimize the potential role of viral proteins in modulating cell death, these recombinant viruses encode deletions of the 78 kDa/NSm genes (20). The recombinant viruses were designated as rMP12-rLuc, rMP12-ORONSs, rMP12-ORONSs-N-Flag, rMP12-ORONSs-C-Flag, and rMP12-ORONSs-SF. They were generated by co-transfecting BHK/T7-9 cells with plasmids encoding the genomic S-segment [pProT7-vS(+)-rLuc, pProT7-vS(+)-ORONSs, pProT7-vS(+)-ORONSs-N-Flag, pProT7-vS(+)-ORONSs-C-Flag, pProT7-vS(+)-ORONSs-SF], the genomic M- and L-segments [pProT7-vM(+)∆NSm and pProT7-vL(+)], along with plasmids expressing the MP-12 N, L, and GnGc proteins (pT7-IRES-vN, pT7-IRES-vL, and pCAGGS-vG) (20–22). Additionally, we generated an MP-12 strain expressing the MP-12 NSs protein fused with a C-terminal Flag-tag (rMP12-RVFNSs-C-Flag) to compare the accumulation patterns of OROV NSs and RVFV NSs. All cells and viruses used in this study were verified as mycoplasma-free through testing conducted at the UTMB Next Generation Sequencing or Tissue Culture Core Facilities.

### Plasmids

The pProT7-vS(+)-ORONSs, pProT7-vS(+)-ORONSs-N-Flag, pProT7-vS(+)-ORONSs-C-Flag, and pProT7-vS(+)-ORONSs-SF plasmids were generated by cloning the NSs open reading frame (ORF) of the OROV MD023 strain into pProT7-vS(+), replacing the original MP-12 NSs gene ORF. Similarly, pProT7-vS(+)-RVFNSs-Flag was generated by replacing the NSs ORF with an NSs ORF fused to a Flag-tag at the C-terminus. The plasmids pProT7-vM(+)∆NSm, pProT7-vL(+), pT7-IRES-vN, pT7-IRES-vL, and pCAGGS-vG have been previously reported (20, 21).

### Analysis of NSs amino acid sequences

The amino acid sequences of NSs proteins from OROV MD023 strain (GenBank no. AF164550), AM0088/Brazil/2024 (GenBank no. PP992525), BeAn19991 strain (GenBank no. KP052852), Shamonda virus An5550 strain (GenBank no. NC_018464.1), SBV BH80/11-4 strain (GenBank no. HE649914.1), and La Crosse virus LACV/human/1978 strain (GenBank no. EF485033) were aligned using CLC Genomics Workbench 7.5.5. Predictions of nucleolar localization sequences within NSs proteins of the SBV BH80/11-4 strain and OROV MD023 strain were performed using the NoD web server (23).

### Western blot analysis

Cells were lysed in 2× Laemmli sample buffer (Bio-Rad) containing 5% 2-mercaptoethanol and heated at 100°C for 10 minutes. Protein concentrations were measured using the Qubit Protein Assay Kit (Invitrogen) following the manufacturer’s instructions. A total of 800 ng of protein was loaded onto an SDS-PAGE gel. Western blotting was performed using Immobilon-P polyvinylidene fluoride membranes (Millipore), and signal detection was carried out with ECL Prime Western Blotting Reagent (Cytiva Life Sciences).

### Indirect fluorescence assay

Vero cells in 24-well plates were fixed with a 1:1 methanol-acetone solution at room temperature for 15 minutes. The cells were rehydrated with phosphate-buffered saline (PBS) and blocked with PBS containing 0.5% bovine serum albumin at 37°C for 1 hour. Following blocking, cells were incubated with primary antibodies (anti-Flag or anti-B32), washed with PBS, and then incubated with Alexa Fluor 488- or 594-conjugated anti-IgG (H+L) antibodies for signal detection. Nuclei were stained with DAPI (4′,6-diamidino-2-phenylindole; Thermo Fisher Scientific). Stained cells were visualized using an Olympus IX73 microscope equipped with a DP71 camera (Olympus America, Center Valley, PA), and images were captured and processed using Olympus cellSens software (version 3.2).

### Antibodies

The following antibodies were used in this study: anti-Flag tag (DYKDDDDK) rabbit monoclonal antibody (Cell Signaling Technology, #14793), anti-Flag M2 mouse monoclonal antibody (Millipore Sigma, F3165), anti-Histone H3 rabbit polyclonal antibody (Cell Signaling Technology, #9715), anti-β-actin mouse monoclonal antibody (Proteintech, #66009-1-Ig), anti-GAPDH mouse monoclonal antibody (Invitrogen, MA5-15738), anti-OROV mouse immune ascitic fluid (ATCC: VR-1228AF), anti-RVFV mouse immune ascitic fluid (WRCEVA, UTMB), anti-phosphorylated RNAP II CTD (Ser5) mouse monoclonal antibody (Thermo Fisher Scientific, #91119), anti-phosphorylated RNAP II CTD (Ser2) rabbit monoclonal antibody (Thermo Fisher Scientific, MA5-33187), anti-RNAP RPB1 mouse monoclonal antibody (Novus Biologicals, NB200-598), and anti-B23 (nucleophosmin 1 [NPM1]) mouse monoclonal antibody (Millipore Sigma, B0556). Additionally, we used a custom anti-OROV NSs peptide (Cys-SSTKRRPKMSYVRHRGPW) rabbit polyclonal antibody (Biomatik).

### Separation of nuclear and cytoplasmic fractions

Vero cells were trypsinized, collected into 15 mL tubes, and washed with cold PBS. Cells were then treated with PBS containing 1% Triton X-100 for 5 minutes on ice, followed by centrifugation at 10,000 × *g* for 5 minutes at 4°C. The supernatant (cytoplasmic fraction) and pellet (nuclear fraction) were collected separately, with the nuclear fraction washed once with cold PBS. Samples were mixed with 2× Laemmli sample buffer (Bio-Rad) containing 5% 2-mercaptoethanol and heated at 100°C for 10 minutes before western blot analysis.

### Quantification of mRNA, rRNA, and viral RNA by qPCR

Vero cells were mock-infected or infected with wt OROV, rMP12-ORONSs, or rMP12-ORONSs-SF at a multiplicity of infection (MOI) of 5. Total RNA was extracted at 16 hours post-infection (hpi) using the RNeasy Mini Kit (Qiagen), following the manufacturer’s instructions. First-strand cDNA was synthesized from 180 ng of total RNA using iScript Reverse Transcriptase (Bio-Rad). qPCR was then performed in a 20 µL reaction using the SsoAdvanced Universal Probes Supermix (Bio-Rad) and cDNA corresponding to 18 ng of input RNA. Reactions were run on a Mic qPCR Cycler (4-channel) with the following cycling conditions: initial denaturation at 98°C for 5 minutes; 40 cycles of 98°C for 15 seconds and 60°C for 45 seconds; followed by a final denaturation step at 98°C for 10 minutes. The PCR reaction targeted the ORF of Vero cell β-actin using the forward primer (Vero-actin-F: 5′- AAG GAT TCA TAT GTG GGC GAT G -3′), the reverse primer (Vero-actin-R: 5′- GTA GAA GGT GTG GTG CCA GA -3′), and Taqman probe (Taq-Vero-actin: 5′FAM- CTC ACC CTG AAG TAC CCC ATC GAG CAC -3′ZEN/IBFQ). The PCR reaction targeted the ORF of OROV NSs gene using the forward primer (ORO-NSs-F: 5′- GGC ATT TGA AGC TAG ATA CGG A -3′), the reverse primer (ORO-NSs-R: 5′- GGC ACT GGA TTC GAC TGG -3′), and Taqman probe (Taq-ORO-NSs: 5′FAM- TAA GAC ATC GAG GCC CAT GGT TGA CC -3′ZEN/IBFQ). 18S rRNA levels were also measured using the Eukaryotic 18S rRNA Endogenous Control Kit (Applied Biosystems, #4319413E). Levels of β-actin mRNA, 18S rRNA, and OROV NSs RNA (S genomic RNA, N and NSs mRNA for wt OROV, S genomic RNA, NSs mRNA for rMP12-ORONSs or rMP12-ORONSs-C-Flag) were separately measured by qPCR. Levels of β-actin mRNA and 18S rRNA were measured by qPCR using gene-specific TaqMan probes. Samples from mock-infected, wt OROV-infected, rMP12-ORONSs-infected, and rMP12-ORONSs-SF-infected Vero cells were analyzed using the same probe sets. Due to the impact of OROV NSs on host transcription, which might affect mRNA or rRNA levels, normalization to endogenous reference genes was not performed. Instead, equal amounts of total RNA were used for cDNA synthesis to ensure consistent input across samples. All qPCR reactions were performed in technical triplicate. ΔCq values were calculated by subtracting the representative Cq value from the mock-infected samples from each Cq value of the infected samples. For OROV NSs RNA, mock-infected samples showed no amplification; therefore, ΔCq values were calculated using a representative Cq value from the wt OROV samples. Relative quantification of gene expression was then determined by converting these ΔCq values into fold changes relative to the mock control using the formula 2^−ΔCq^. The integrity of 28S and 18S rRNA was assessed by electrophoresing 500 ng of total RNA on a 1% agarose gel stained with SmartGlow Loading Dye (Electron Microscopy Sciences).

### Analysis of cellular nascent RNA synthesis

Analysis of nascent RNA synthesis in mock or infected cells was performed as described previously (24). Briefly, cells were either mock-infected or infected with rMP12-rLuc, rMP12-ORONSs, or wt OROV at an MOI of 5. At 15 hpi, cells were treated with 0.5 mM 5-ethynyluridine (5-EU; Berry & Associates) for 1 hour before harvesting at 16 hpi. A control well was treated with 5 µg/mL actinomycin D (ActD) concurrently with 5-EU to inhibit cellular RNA synthesis. After harvesting, those cells were fixed in 4% paraformaldehyde for 30 minutes, permeabilized with 0.2% Triton X-100 in PBS for 10 minutes, and subjected to click chemistry to detect nascent RNA incorporating 5-EU. This was performed using 200 nM Alexa Fluor 647-azide (Invitrogen) in 100 mM Tris (pH 8.5) containing 1 mM CuSO_4_ and 100 mM ascorbic acid. Viral protein expression was detected by staining with a mouse polyclonal antibody against RVFV or OROV, followed by an Alexa Fluor 488-labeled secondary antibody. Finally, cells were analyzed by fluorescence-activated cell sorting analysis using an LSRII Fortessa instrument (Becton Dickinson).

### Proteasomal inhibitor treatment

To evaluate RNAP II RPB1 stabilization upon proteasomal inhibition, Vero cells were either mock-infected or infected with rMP12-ORONSs or wt OROV at an MOI of 5. At 4 hpi, cells were treated with 10 µM MG-132 (M7449, Millipore Sigma) or DMSO, and lysates were collected at 16 hpi for analysis by western blotting. 

### Statistical analysis

The results were analyzed using one-way or two-way analysis of variance (ANOVA) followed by Tukey’s multiple comparisons test, performed using GraphPad Prism 8.4.3 (GraphPad Software Inc., San Diego, CA).

## RESULTS

### Oropouche virus NSs protein shares similarity with Schmallenberg virus NSs and localizes to the nucleus

**Fig 1:**
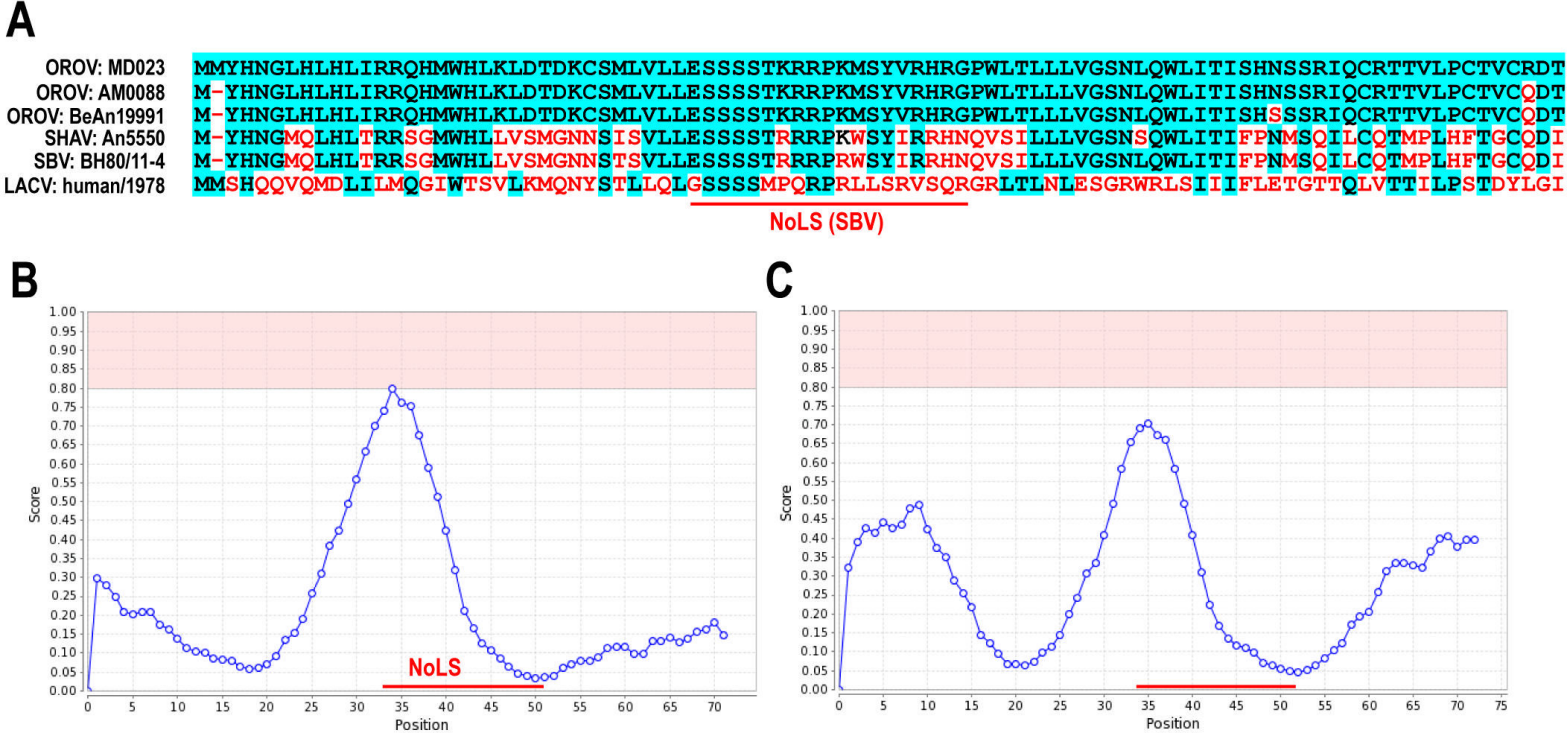
Sequence similarity of NSs proteins between Oropouche virus and Schmallenberg virus. (**A**) Alignment of NSs protein sequences from the OROV MD023 strain (GenBank no. AF164550), AM0088/Brazil/2024 (GenBank no. PP992525), BeAn19991 strain (GenBank no. KP052852), Shamonda virus An5550 strain (GenBank no. NC_018464.1), SBV BH80/11-4 strain (GenBank no. HE649914.1), and La Crosse virus LACV/human/1978 strain (GenBank no. EF485033). Conserved amino acids are highlighted with a blue background, while nonconserved amino acids are marked in red. (**B–C**) Prediction of nucleolar localization sequences within the NSs proteins of the SBV BH80/11-4 strain (**B**) and OROV MD023 strain (**C**) using the NoD web server. The predicted NoLS for SBV, as identified by Gouzil et al. (18), the corresponding NoLS-like region in OROV NSs protein is indicated by a red line.

This study characterized the NSs functions of the MD023 strain, a genotype II virus isolated in Peru in 1996, with an LD_50_ of 45 in hamsters (25). The amino acid sequence of the MD023 NSs protein showed 96.7% identity with the OROV prototype strain BeAn19991 and 97.8% identity with the OROV AM0088 strain, which was isolated during the 2024 outbreak in Brazil (Fig. 1A). Additionally, the MD023 NSs protein shared 57.6% identity with the SBV NSs protein (Fig. 1A). Previous studies showed that SBV NSs proteins localize to the nucleoli via a putative nucleolar localization signal (NoLS: Fig. 1A and B), reorganize their distribution, and lead to the degradation of host RNAP II RPB1 (17, 18). Similarly, nucleolar reorganization was observed in cells infected with Shamonda virus, a member of the Simbu group (18). In the present study, comparative sequence analysis of SBV and OROV NSs revealed similarities in their predicted NoLS (Fig. 1B and C). Based on this, we hypothesized that the OROV NSs protein may also localize to the nucleoli.

### The Oropouche virus NSs protein colocalizes with the nucleophosmin 1

**Fig 2:**
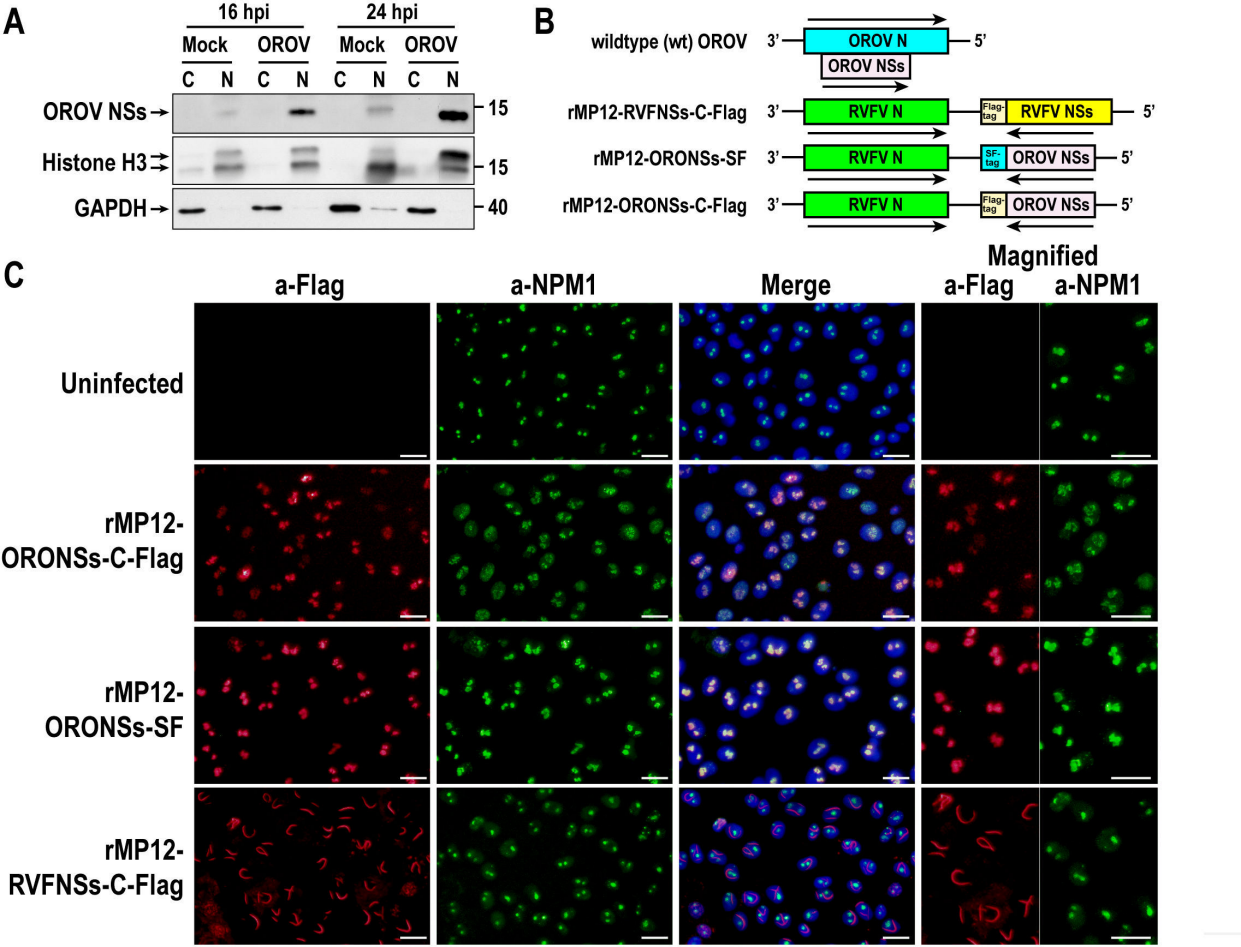
Nuclear localization of Oropouche virus NSs protein. (**A**) Western blot analysis of nuclear and cytoplasmic fractions from OROV-infected cells. Vero cells were either mock-infected or infected with wild-type (wt) OROV at an MOI of 5. Cells were collected at 16 and 24 hpi, and nuclear and cytoplasmic fractions were separated. OROV NSs protein was detected using an anti-OROV NSs peptide antibody. Histone H3 and GAPDH were used as nuclear and cytoplasmic markers, respectively. (**B**) Schematic representation of the S-segments of wt OROV, rMP12-ORONSs-C-Flag, rMP12-ORONSs-SF, and rMP12-RVFNSs-C-Flag. Arrows indicate the direction of mRNA transcription. (**C**) Vero cells were mock-infected or infected with rMP12-ORONSs-C-Flag, rMP12-ORONSs-SF, or rMP12-RVFNSs-C-Flag at an MOI of 5 and fixed at 16 hpi. Cells were double-stained with anti-NPM1 (green) and anti-Flag (red) antibodies. Nuclei were stained with DAPI. Merged images are also shown. Bars represent 25 µm.

We next examined the cellular localization of the OROV NSs protein. Vero cells were either mock-infected or infected with the wild-type OROV MD023 strain at an MOI of 5. Cytoplasmic and nuclear fractions were then prepared, and the presence of OROV NSs proteins was assessed by western blot using specific antibodies. As shown in Fig. 2A, OROV NSs proteins were predominantly detected in nuclear lysates both at 16 and 24 hpi, indicating the nuclear localization of NSs proteins during the infection. Next, we attempted to visualize OROV NSs protein accumulation in the nucleolus using an anti-NSs antibody in an indirect immunofluorescence assay (IFA). However, while this antibody was suitable for western blot, it did not work for IFA. To overcome this limitation, we generated the recombinant RVFV MP-12 strain (rMP-12) in which the OROV NSs gene replaced the RVFV NSs gene in the S-segment to facilitate the visualization of OROV NSs nuclear accumulation (Fig. 2B). To facilitate the detection of OROV NSs by IFA, either a Flag tag or a Strep-Flag (SF) tag was added to the C-terminus of OROV NSs. The resulting viruses were designated rMP12-ORONSs-C-Flag and rMP12-ORONSs-SF, respectively. For comparison, a Flag-tag was also added to the C-terminus of the RVFV rMP-12 NSs protein (rMP12-RVFNSs-C-Flag). We did not use a plasmid-mediated approach because a previous study suggested that the SBV NSs protein, which is similar to the OROV NSs protein, inhibits RNAP II-mediated transcription from plasmid-based constructs (17). Vero cells were mock-infected or infected with rMP12-ORONSs-C-Flag, rMP12-ORONSs-SF, or rMP12-RVFNSs-C-Flag at an MOI of 5. Cells were fixed at 16 hpi, and the localization of NPM1, a nucleolar protein, and the NSs proteins was examined. As shown in Fig. 2C, the OROV NSs-Flag expressed from rMP12-ORONSs-C-Flag and the OROV NSs-SF expressed from rMP12-ORONSs-SF displayed a nucleolar distribution pattern closely resembling that of NPM1, suggesting that the OROV NSs protein colocalizes with NPM1. While cells infected with rMP12-ORONSs-SF exhibited a punctate NPM1 distribution consistent with normal nucleolar localization, those infected with rMP12-ORONSs-C-Flag showed a less distinct and disorganized NPM1 pattern within the nucleus, along with increased NPM1 accumulation in the nucleoplasm, indicating nucleolar disruption and redistribution of NPM1. Meanwhile, RVFV NSs-Flag exhibited its characteristic filamentous structures, as previously reported (26, 27). RVFV NSs-Flag did not co-localize with NPM1 but led to increased NPM1 abundance in the nucleoplasm.

### Wild-type OROV infection induces the redistribution of nucleophosmin 1 from the nucleoli

**Fig 3:**
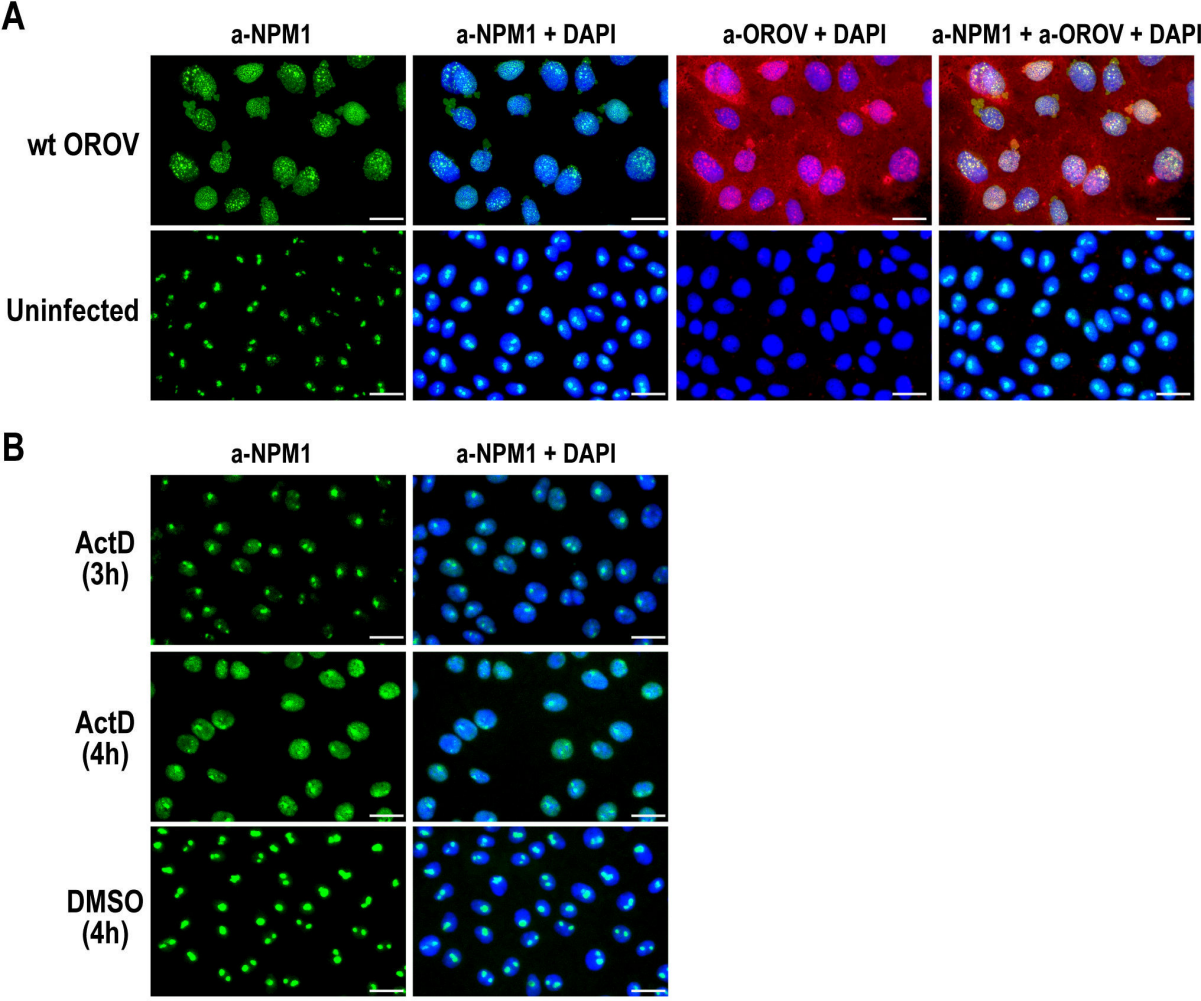
Translocation of NPM1 in wild-type OROV-infected cells. (**A**) Vero cells were mock-infected or infected with wild-type (wt) OROV at an MOI of 5 and fixed at 16 hpi. Cells were stained with anti-NPM1 (green) and anti-OROV ascites (red). Nuclei were counterstained with DAPI (blue). Merged images are also shown. (**B**) Vero cells were treated with DMSO (mock) for 4 hours, or 5 µg/µL ActD for 3 or 4 hours. Cells were stained with anti-NPM1 (green) and DAPI (blue). Merged images are also shown. Bars represent 25 µm.

We next investigated whether the infection with wt OROV induces the redistribution of NPM1 from the nucleolus. Vero cells were mock-infected or infected with wt OROV at an MOI of 5. Cells were fixed at 16 hpi and stained with anti-NPM1 antibody and anti-OROV ascitic fluid for IFA. As shown in Fig. 3A, cells infected with wt OROV exhibited redistribution of NPM1, which no longer displayed its characteristic punctate nucleolar localization. Instead, NPM1 adopted a granular pattern within the nucleus and along the nuclear membrane, with occasional extension into the perinuclear region. The anti-OROV ascitic fluid appeared to stain both the cytoplasm and the nucleus. Although the diffuse cytoplasmic staining likely corresponds to the N protein, the granular nuclear staining partially overlaps with NPM1, suggesting that the anti-OROV ascitic fluid may also detect OROV NSs proteins. The pronounced redistribution of NPM1 from the nucleoli observed in OROV-infected cells indicates nucleolar stress, a condition that can be triggered by various stimuli, including inhibition of rRNA synthesis. As shown in Fig. 3B, Vero cells treated with 5 µg/mL ActD exhibited redistribution of NPM1 at 4 hours after treatment; however, the staining pattern was not granular and differed from that observed in wt OROV-infected cells. Treatment of cells with a high dose of ActD inhibits cellular RNA synthesis by intercalating into DNA and thereby blocking the activity of all three RNA polymerases (28, 29). This transcriptional inhibition likely induces nucleolar stress, leading to the redistribution of NPM1 from its typical nucleolar localization into the nucleoplasm.

### OROV NSs protein expression induces general transcriptional suppression

**Fig 4:**
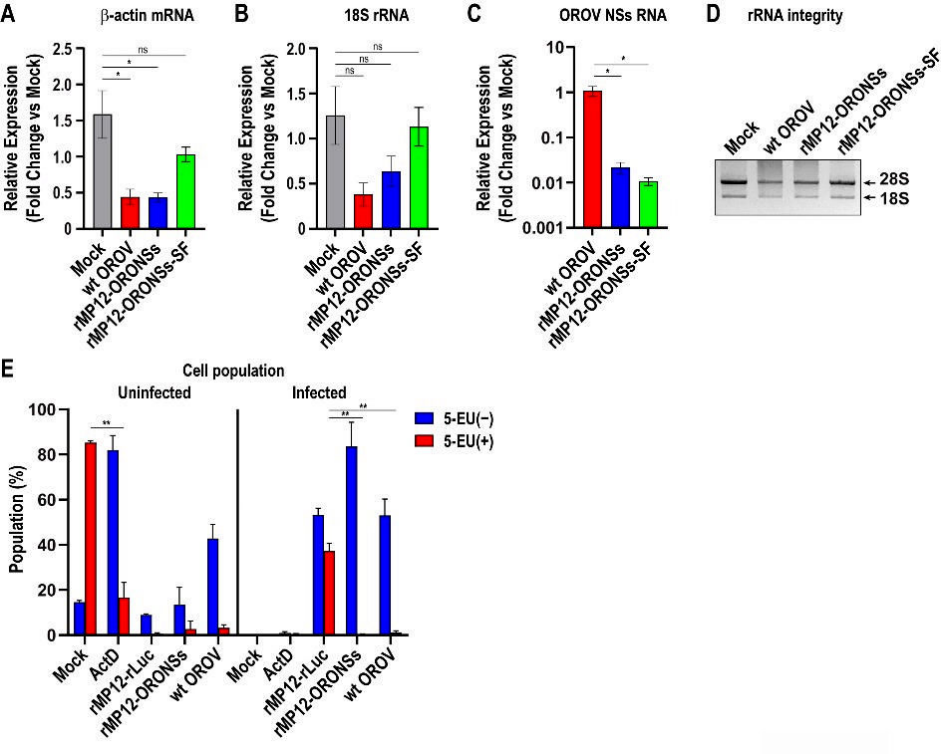
Effects of OROV infection on host mRNA/rRNA expression, rRNA integrity, and nascent RNA synthesis. (**A–C**) Vero cells were mock-infected or infected with wild-type (wt) OROV, rMP12-ORONSs, or rMP12-ORONSs-SF at an MOI of 5. At 16 hpi, the relative expression levels of β-actin mRNA (**A**), 18S rRNA (**B**), and OROV RNA corresponding to the NSs ORF (**C**) were quantified by qPCR using 18 ng of total RNA per reaction. The graphs show the mean ± standard error. (**D**) Total RNA was resolved on a 1% agarose gel to visualize 28S and 18S rRNA integrity with SmartGlow Loading Dye. (**E**) Vero cells were mock-infected or infected with the indicated viruses at an MOI of 5. At 15 hpi, cells were incubated with 5-EU for 1 hour and harvested at 16 hpi. A control well was treated with 5 µg/mL ActD concurrently with 5-EU to inhibit cellular RNA synthesis. Incorporated 5-EU was detected via click chemistry using Alexa Fluor 647-azide. Infected cells were identified using anti-OROV or anti-RVFV mouse ascitic fluid, followed by an Alexa Fluor 488-labeled secondary antibody. The graph shows the mean ± standard deviation of the percentage of 5-EU-positive and 5-EU-negative cells within uninfected and infected populations, based on three independent experiments. Asterisks indicate statistical significance (*P < 0.01, one-way ANOVA for A–C; **P < 0.0001, two-way ANOVA for E). ns, not significant.

Since NPM1 redistribution was observed in cells infected with wt OROV or rMP12-ORONSs-C-Flag, but not in those infected with rMP12-ORONSs-SF, we next investigated whether cellular RNA levels were affected during virus infection. Vero cells were mock-infected or infected with wt OROV, rMP12-ORONSs, or rMP12-ORONSs-SF at an MOI of 5. Total RNA was extracted at 16 hpi, and the relative expression levels of β-actin mRNA, 18S rRNA, and OROV NSs RNA were quantified by qPCR using equal amounts of input RNA. As shown in Fig. 4A and B, cells infected with wt OROV showed a significant reduction in β-actin mRNA levels (3.6-fold; *P* = 0.0098) and a non-significant decrease in 18S rRNA levels (3.3-fold; *P* = 0.0858) compared to mock-infected cells. Similarly, infection with rMP12-ORONSs led to a significant reduction in β-actin mRNA (3.6-fold; *P* = 0.0095) and a non-significant decrease in 18S rRNA (2.0-fold; *P* = 0.2661). In contrast, cells infected with rMP12-ORONSs-SF exhibited only a modest, non-significant reduction in β-actin mRNA (1.5-fold; *P* = 0.2230) and no appreciable change in 18S rRNA levels (1.1-fold; *P* = 0.9764). These findings indicate that β-actin mRNA expression, but not 18S rRNA, was significantly reduced in cells infected with wt OROV or rMP12-ORONSs. The qPCR using a probe specific to the OROV NSs ORF revealed significantly lower levels of viral RNA in cells infected with rMP12-ORONSs (50-fold reduction; *P* = 0.0062) and rMP12-ORONSs-SF (101-fold reduction; *P* = 0.0059) compared to cells infected with wt OROV. The NSs ORF overlaps with the N ORF on the S segment of wt OROV, allowing the probe to detect not only the genomic S-segment RNA but also both N and NSs mRNAs. In contrast, rMP12-ORONSs and rMP12-ORONSs-SF express OROV NSs from an ambisense orientation, such that the probe detects only the S-segment RNA and NSs mRNA, but not N mRNA. Therefore, differences in probe target specificity likely contribute to the lower apparent levels of NSs RNA detected in the recombinant virus-infected cells. We also analyzed the 28S and 18S rRNA from total RNA samples by 1% agarose gel electrophoresis (Fig. 4D). The 28S and 18S rRNA bands in rMP12-ORONSs-SF-infected cells had intensities comparable to those in mock-infected controls. In contrast, cells infected with wt OROV or rMP12-ORONSs exhibited slightly reduced 28S and 18S rRNA band intensities. Additionally, in these samples, a diffuse, darker background smear appeared above the 18S rRNA band, corresponding to RNA species of similar or higher molecular weight. This smear likely represents RNA degradation products or abnormal RNA processing intermediates, as no discrete additional bands were observed.

We next investigated whether the expression of OROV NSs can suppress cellular nascent RNA synthesis. Vero cells were either mock-infected or infected with wt OROV or rMP12-ORONSs at an MOI of 5. Cells infected with rMP12-rLuc served as a control, lacking the OROV NSs expression. The uridine analog 5-EU was added to cells at 15 hpi, followed by a 1 hour incubation. As a positive control for host RNA synthesis inhibition, uninfected cells were pretreated with 5 µg/mL ActD concurrently with 5-EU. Incorporated 5-EU in nascent RNA was detected using click chemistry, where 5-EU was covalently conjugated to Alexa Fluor 647-azide via copper-catalyzed azide-alkyne cycloaddition. Cells were additionally stained with anti-OROV or anti-RVFV antibodies and analyzed by flow cytometry to distinguish four populations: uninfected 5-EU(−), uninfected 5-EU(+), infected 5-EU(−), and infected 5-EU(+). As shown in Fig. 4E, mock-infected cells exhibited robust 5-EU incorporation, with 85.3% classified as 5-EU(+), indicating active transcription. In contrast, ActD treatment significantly reduced 5-EU incorporation, resulting in only 16.7% 5-EU(+) cells (*P* < 0.0001 vs mock). Cells infected with rMP12-rLuc contained 53.2% 5-EU(−) and 37.2% 5-EU(+) populations, whereas rMP12-ORONSs-infected cells consisted of 83.5% 5-EU(−) and only 0.3% 5-EU(+). Similarly, wt OROV-infected cells displayed 53% 5-EU(−) and 1.1% 5-EU(+). Accordingly, the proportion of infected cells classified as 5-EU(+) was significantly reduced in those infected with wt OROV or rMP12-ORONSs at 16 hpi (*P* < 0.0001 vs rMP12-rLuc). The results indicate that nascent cellular RNA synthesis is impaired in cells infected with wt OROV or rMP12-ORONSs.

### Phosphorylation of the RNA polymerase II C-terminal domain is altered during OROV NSs protein expression

**Fig 5:**
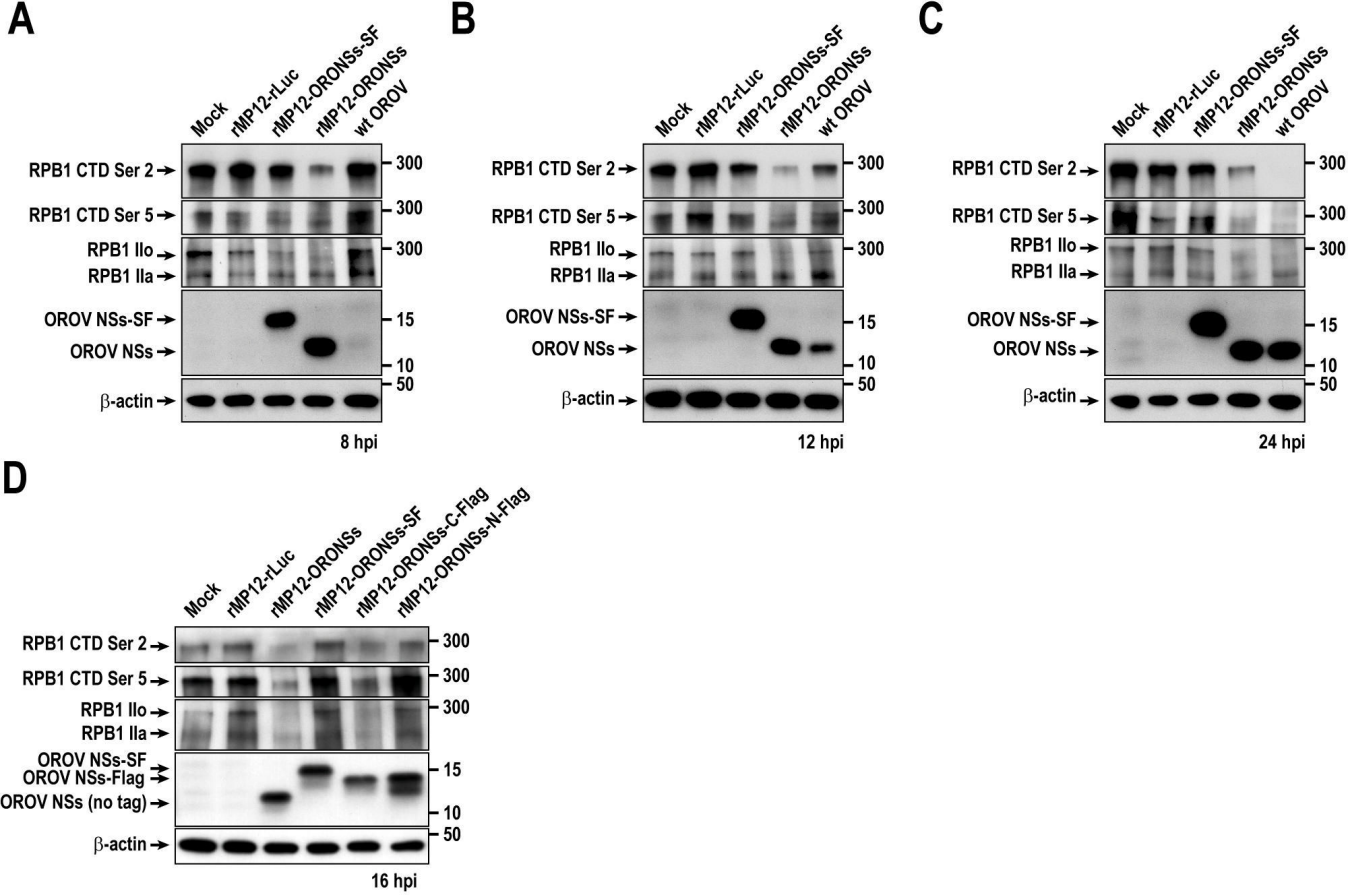
Altered phosphorylation status and stability of the RNA polymerase II RPB1 subunit upon expression of Oropouche virus NSs protein. (**A–C**) Western blot analysis of total cell lysates from Vero cells infected with wild-type (wt) OROV or rMP12 NSs variants. Cells were mock-infected or infected at an MOI of 5 and harvested at 8 hpi (**A**), 12 hpi (**B**), or 24 hpi (**C**). (**D**) Cells were similarly infected and harvested at 16 hpi. The blot shows the hyperphosphorylated (IIo) and hypophosphorylated (IIa) forms of the RNA polymerase II RPB1 subunit, as well as CTD phosphorylation at serine 2 (Ser 2) and serine 5 (Ser 5), are shown. Expression of OROV NSs, as well as Flag- or SF-tagged NSs proteins, was detected using an anti-OROV NSs peptide antibody.

We next examined the changes in RNAP II RPB1 CTD phosphorylation during the expression of OROV NSs proteins. In addition to wt OROV infection, we assessed OROV NSs expression using recombinant MP-12 viruses: rMP12-ORONSs and rMP12-ORONSs-SF. As a control lacking OROV NSs expression, we used rMP12-rLuc. As shown in Fig. 5A through C, cells infected with wt OROV did not exhibit detectable levels of NSs protein at 8 hpi but began accumulating NSs protein by 12 hpi, with its abundance increasing further by 24 hpi. Meanwhile, cells infected with rMP12-ORONSs or rMP12-ORONSs-SF accumulated detectable NSs proteins as early as 8 hpi. Compared to mock-infected cells, those infected with wt OROV exhibited a detectable reduction in phosphorylation at serine 2 and serine 5 of the CTD by 12 hpi, which became nearly undetectable by 24 hpi. An analysis of total RPB1 protein revealed that the hyperphosphorylated IIo form, but not the hypophosphorylated IIa form, was nearly undetectable in wt OROV-infected cells. Meanwhile, cells infected with rMP12-ORONSs exhibited a detectable reduction in CTD phosphorylation at serine 2 and serine 5 as early as 8 hpi. Although phosphorylated CTD remained detectable at 12 and 24 hpi, its levels were markedly reduced. Analysis of total RPB1 protein showed a decrease in the IIo form by 8 hpi, while the IIa form remained detectable at 24 hpi. Cells infected with rMP12-ORONSs-SF did not show a detectable reduction in RPB1 or phosphorylation at serine 2 and serine 5 of the CTD by 24 hpi. These results indicate that infection with wt OROV or rMP12-ORONSs leads to a predominant reduction in the RPB1 IIo form, coinciding with the accumulation of OROV NSs protein and a marked decrease in serine 2 and serine 5 phosphorylation of the RPB1 CTD. In contrast, rMP12-ORONSs-SF infection did not affect the RPB1 IIo form or CTD phosphorylation, suggesting that addition of the SF-tag to the C-terminus abolishes the biological function of OROV NSs in suppressing RPB1.

Additionally, rMP12-ORONSs-C-Flag and rMP12-ORONSs-N-Flag were tested to evaluate the impact of placing the Flag tag at the C-terminus or N-terminus, respectively. As shown in Fig. 5D, Vero cells infected with rMP12-ORONSs-C-Flag, but not with rMP12-ORONSs-N-Flag, exhibited a reduction in RPB1 CTD phosphorylation at serine 2 and serine 5. Infection with rMP12-ORONSs-N-Flag resulted in the accumulation of two distinct NSs bands, suggesting that the N-terminal Flag tag compromises the stability of the OROV NSs protein in infected cells.

### Post-translational degradation contributes to the reduction of RPB1 during Oropouche virus infection

**Fig 6:**
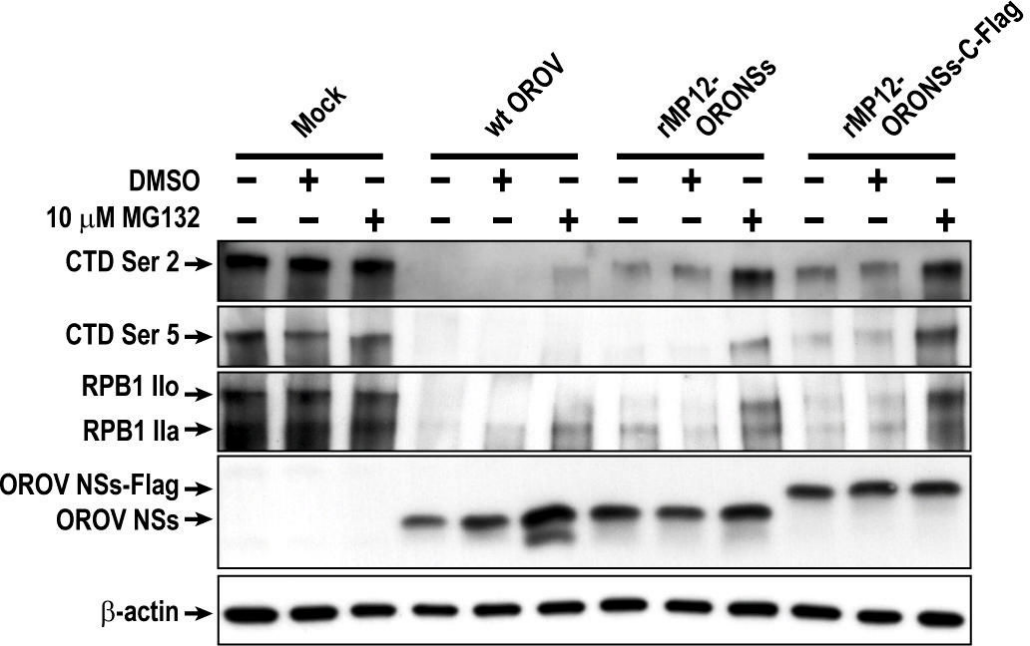
Stabilizing effect of the proteasomal inhibitor MG-132 on the RNA polymerase II RPB1 subunit during Oropouche virus NSs protein expression. Vero cells were either mock-infected or infected with wild-type (wt) OROV, rMP12-ORONSs, or rMP12-ORONSs-C-Flag at an MOI of 5. A 10 µM concentration of MG-132 was added to the culture at 4 hpi. Cells were harvested at 16 hpi, and the levels of the RNAP II RPB1 subunit, as well as its phosphorylated forms at serine 2 (Ser 2) or serine 5 (Ser 5) within the CTD, were analyzed. OROV NSs protein was detected using an anti-OROV NSs peptide antibody.

We examined whether the reduction of RNAP II RPB1 was affected by the proteasome inhibitor MG-132. Vero cells were either mock-infected or infected with wt OROV, rMP12-ORONSs, or rMP12-ORONSs-C-Flag at an MOI of 5. At 4 hpi, 10 µM MG-132 was added to the culture medium, and cells were incubated until 16 hpi. As shown in Fig. 6, wt OROV-infected cells exhibited a reduction in both the IIa and IIo forms of RPB1 at 16 hpi in the absence of MG-132, with the IIo form being only faintly detectable. Following MG-132 treatment, western blot analysis revealed an increase in the IIo form and restored detection of CTD serine 2 phosphorylation in wt OROV-infected cells. Meanwhile, Vero cells infected with rMP12-ORONSs or rMP12-ORONSs-C-Flag at 16 hpi also exhibited a reduction in both the IIa and IIo forms of RPB1 in the absence of MG-132. Following MG-132 treatment, the levels of both the IIa and IIo forms of RPB1, as well as CTD phosphorylation at serine 2 and serine 5, were restored. These results indicate that RPB1 instability in cells infected with rMP12-ORONSs or rMP12-ORONSs-C-Flag is driven by proteasome-mediated degradation. In contrast, the partial restoration of RPB1 levels, along with CTD phosphorylation at serine 2, in wt OROV-infected cells following MG-132 treatment, suggests that additional OROV proteins may contribute to the enhanced reduction of RPB1 in these cells.

## DISCUSSION

This study demonstrated that infection with the OROV MD023 strain leads to nuclear accumulation of NSs and induces redistribution of NPM1 from the nucleolus. The infection with wt OROV suppressed nascent RNA synthesis and significantly reduced β-actin mRNA levels by 16 hpi, accompanied by a non-significant decrease in 18S rRNA levels. This transcriptional suppression was most likely associated with a decrease in RNAP II RPB1 protein levels. Treatment with MG-132 partially stabilized RPB1, indicating involvement of proteasome-mediated degradation. The partial restoration of RPB1 following MG-132 treatment also suggests that its reduction may occur, at least in part, through proteasome-independent mechanisms. A previous study showed that recombinant OROV lacking either NSs or NSm protein could induce host translational shutoff (19). Therefore, the lack of full RPB1 stabilization by MG-132 in wt OROV-infected cells may be due to a reduced protein turnover rate, resulting from cellular conditions that inhibit host protein synthesis, independently of NSs or NSm proteins (19). Meanwhile, expression of OROV NSs from a recombinant MP-12 strain induced a predominant reduction in the RPB1 IIo form and a partial decrease in CTD phosphorylation at serine 2 and serine 5. Furthermore, the levels of RPB1 and its CTD phosphorylation at serine 2 and serine 5 were largely restored following MG132 treatment. Since RPB1 reduction was not observed in cells infected with rMP12-rLuc, which lacks OROV NSs expression, these findings suggest that OROV NSs contributes to the proteasomal degradation of RPB1, predominantly affecting the IIo form.

Expression of OROV NSs from a recombinant MP-12 strain confirmed that NSs localize to the nucleolus, co-localize with NPM1, and likely contribute to NPM1 redistribution. Interestingly, NSs expressed without a tag or with a C-terminal Flag tag reduced RPB1 levels, whereas NSs with a C-terminal SF-tag or an N-terminal Flag tag did not affect RPB1. Notably, NSs-SF still accumulated in nucleoli and co-localized with NPM1 without inducing its redistribution. Cells infected with rMP12-ORONSs-SF failed to inhibit CTD phosphorylation or promote RPB1 degradation. The OROV NSs-SF is a fusion protein bearing a C-terminal SF-tag. Previous studies have shown that mutations in the C-terminal region of the SBV NSs protein (e.g., I67A, I69A, or P82A) abolish its ability to inhibit host transcription, highlighting the critical role of this region in NSs function (17). This suggests that the C-terminal SF-tag may interfere with essential interactions between OROV NSs and yet-unidentified host factors required for transcriptional inhibition. In contrast, cells infected with rMP12-ORONSs-C-Flag promoted the RPB1 degradation, despite the presence of a C-terminal Flag tag. The SF tag consists of 20 amino acids, including a linker, while the C-Flag tag contains only eight amino acids. These differences in size and composition likely influence the biological activity of the NSs protein. Together, these findings suggest that both C-Flag- or SF-tagged NSs variants may serve as useful tools for dissecting the molecular mechanisms underlying nucleolar disruption and host transcriptional shutoff.

LACV infection has been shown to specifically induce the degradation of the RNAP II RPB1 IIo form (16). It has been proposed that RPB1 degradation may be triggered by stalled RNAP II elongation, particularly in response to DNA damage (30, 31). However, a follow-up study demonstrated that Elongin C functions as an NSs cofactor in the ubiquitin ligase complex, facilitating the proteasomal degradation of RPB1 (32). Notably, Elongin C co-localizes with nucleolin, a nucleolar protein, whereas wt LACV infection induces the redistribution of Elongin C from the nucleoli (32). Similarly, SBV infection in HEK-293T cells has been reported to cause RNAP II RPB1 degradation, affecting both the IIo and IIa forms, a process that can be stabilized by MG-132, a proteasomal inhibitor (17). Although the precise mechanism of SBV NSs-mediated RPB1 degradation remains unclear, SBV NSs proteins also co-localize with nucleolar proteins such as NPM1, leading to its redistribution from the nucleolus to the nucleoplasm (18). This translocation is likely caused by nucleolar stress, including the inhibition of RNAP II. Our study further confirmed that NPM1 translocation occurs in cells infected with either wt OROV or rMP12-ORONSs-C-Flag, both of which can inhibit RPB1, but not in cells infected with rMP12-ORONSs-SF, which expresses a nonfunctional NSs. These findings suggest a virulence mechanism similar to that observed in SBV and LACV infections, in which nuclear stress resulting from host RPB1 inhibition contributes to NPM1 redistribution.

Our study showed that cells infected with wt OROV or rMP12-ORONSs, but not those infected with rMP12-ORONSs-SF, exhibited reduced 18S rRNA levels compared to mock-infected cells, although the differences were not statistically significant. Additionally, these infected cells displayed increased RNA smearing above the molecular weight of 18S rRNA, suggesting potential RNA processing defects or degradation. NPM1 is one of the most abundant nucleolar proteins and typically resides in the nucleolus, but it can shuttle between the nucleolus and nucleoplasm in response to nucleolar stress (29). Such stress, and the accompanying redistribution of NPM1, can be triggered by inhibition of RNA polymerase I activity, such as through ActD treatment. Meanwhile, it remains unclear whether OROV NSs directly cause transcriptional inhibition of rRNA. If they do not, the observed inhibition of rRNA synthesis may be a secondary consequence of nuclear stress. However, the downstream effects of nucleolar stress on rRNA synthesis are not yet fully understood. In wt OROV-infected cells, NPM1 exhibited a distinct distribution compared to ActD-treated cells, characterized by a granular pattern within the nucleus and along the nuclear membrane, with occasional extension into the perinuclear region. Further studies are warranted to explore nucleolar remodeling driven by the interaction between NPM1 and OROV NSs.

A previous study predicted a NoLS within SBV NSs at amino acids 33–51 (18). However, the predicted NoLS did not meet the positive predictive value threshold in the NoD web server (23). Despite this, Gouzil et al. (18) demonstrated that SBV NSs protein indeed colocalized with nucleoli. They also found that two close stretches of basic residues, RRR (aa.39–41) and RRH (aa.48–50), play an important role in the nucleolar targeting. Similarly, the OROV NSs protein contains a NoLS-like sequence at amino acids 34–52 with similar basic residues of KRR (aa.40–42) and RHR (aa.49–51), sharing overall 63% identity with its SBV counterpart (18). Interestingly, the LACV NSs protein also targets nucleoli, likely through the interaction with Elongin C (32). The localization of NSs proteins in the nucleoli, followed by nucleolar disruption and RNAP II degradation, likely represents a common virulence mechanism among these pathogenic orthobunyaviruses.

In conclusion, the OROV NSs protein localizes to the nucleolus and suppresses host transcription by promoting the post-translational degradation of the RNAP II RPB1 subunit, primarily the hyperphosphorylated IIo form. In addition, OROV NSs colocalize with NPM1 and induce its redistribution. In contrast, the SF-tagged OROV NSs protein, which retains NPM1 colocalization but lacks the ability to degrade RPB1, does not trigger NPM1 redistribution. These findings suggest that NPM1 redistribution may be associated with nucleolar stress induced by transcriptional suppression.

## Data Availability

The data presented in this study are available on request from the corresponding author.
